# Splenic artery rupture and bleeding following endoscopic retrograde cholangiopancreatography: A case report

**DOI:** 10.1097/MD.0000000000046942

**Published:** 2026-01-09

**Authors:** Zhiqiang Huang, Yunsheng Qin

**Affiliations:** a Department of Hepatobiliary Surgery, Shaoxing Central Hospital, Shaoxing, Zhejiang Province, China; b Department of Hepatobiliary Surgery, The First Affiliated Hospital of Zhejiang University, Hangzhou, Zhejiang Province, China.

**Keywords:** endoscopic retrograde cholangiopancreatography (ERCP), short gastric artery, splenic injury

## Abstract

**Introduction:**

Common complications after endoscopic retrograde cholangiopancreatography (ERCP) include acute pancreatitis, gastrointestinal perforation or bleeding, while splenic artery rupture and bleeding is a very rare complication.

**Main symptoms:**

This study reports a case of splenic artery rupture and bleeding after ERCP, providing reference experience for clinicians. This article describes the case of a 67-year-old patient who underwent ERCP to alleviate jaundice associated with pancreatic head cancer. During the procedure, the patient experienced unexpected hemorrhagic shock.

**Diagnoses, interventions, and outcome:**

The shock was later confirmed as distal splenic artery (short gastric artery) bleeding by digital subtraction angiography, without splenic capsule tearing or subcapsular hematoma. Distal splenic artery branch embolization was performed immediately to avoid splenectomy, and satisfactory results were achieved.

**Conclusion:**

Splenic artery bleeding is an unexpected and extremely rare complication after ERCP and distal splenic artery branch embolization with digital subtraction angiography is a possible treatment approach. This case provides physicians with valuable experience, highlighting the importance of being highly suspicious of such rare complications, and discussing the possible causes and treatment methods for splenic injury following ERCP.

## 1. Introduction

Endoscopic retrograde cholangiopancreatography (ERCP) is widely used in the diagnosis and treatment of hepatobiliary and pancreatic diseases, and has advantages such as minimal trauma, rapid recovery, and safety.^[1]^ Common postoperative complications include acute pancreatitis, gastrointestinal perforation, gastrointestinal bleeding, and biliary tract infections.^[2]^ Splenic injury or splenic artery bleeding is an unexpected and extremely rare complication, with only 34 cases reported worldwide to date.^[1]^ Most cases involve splenic capsule tearing or subcapsular hematoma, and there have been no reports of isolated rupture of the short gastric artery with bleeding. Owing to the lack of specific clinical manifestations in the early stage, it often presents with sudden abdominal pain, which overlaps with post-ERCP abdominal pain symptoms, making early differential diagnosis difficult.^[3]^ Therefore, it is challenging for physicians to observe this complication in clinical practice, delaying timely and effective intervention, which can sometimes lead to fatal outcomes. In these studies, 70.59% (24/34) of patients underwent splenectomy, and only 3 patients underwent interventional treatment.^[1]^ The case discussed in this paper avoided the significant trauma of open surgery and the sequelae of splenectomy through selective splenic artery embolization, confirming the important role of interventional treatment in unexpected splenic injuries.

## 2. Case report

As this study is a case report, ethical approval is not required and informed written consent was obtained from the patient’s guardian for the publication of this case report.

**Figure 1.**
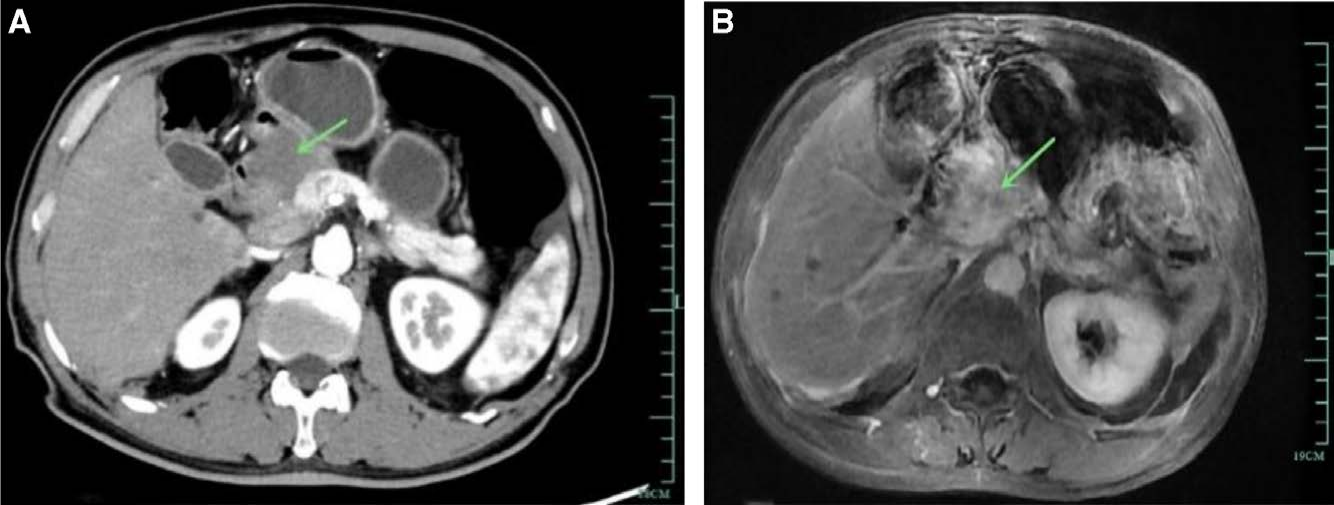
(A) The enhanced CT scan reveals a tumor in the head of the pancreas, with possible invasion of the pylorus and duodenum, and gastric retention with dilation. (B) The enhanced MRI shows tumor invasion of the bile duct. CT = computed tomography, MRI = magnetic resonance imaging.

A 67-year-old female patient with a body mass index of 21.19, who was admitted to the hospital because of jaundice of the skin and sclera accompanied by abdominal discomfort for 20 days, also experienced yellowing of urine, abdominal bloating and discomfort, mainly dull pain, accompanied by nausea and acid reflux, without vomiting, fever, or chills. She had no hypertension or diabetes and no history of surgery. Physical examination revealed skin and scleral jaundice, a soft abdomen, no tenderness or rebound tenderness in the abdomen, and a negative Murphy sign. The tumor markers CA199 was 2187 U/mL, CA125 was 485.8 U/mL. Liver function was as follows: alanine aminotransferase 174 U/L, aspartate aminotransferase 117 U/L, alkaline phosphatase 282 U/L, total bilirubin 11.6 mg/dL, direct bilirubin 11.0 mg/dL, Gamma-glutamyl transferase 342 U/L. Blood routine: white blood cells 6.33 × 10^9^/L, red blood cells 4.6 × 10^12^/L, Hemoglobin 140 g/L, and haematocrit 42.7%. Enhanced abdominal computed tomography (CT) revealed the following: a pancreatic head lesion, invading the antrum of the stomach and the bulb of the duodenum, considering adenocarcinoma; nodular enhancement in segment S5 of the liver, considering metastasis; multiple peritoneal, omental, and mesenteric metastatic implants; local significant enhancement of the gastric wall, thickening of the duodenal wall with stenosis, and possibly tumor involvement. Enhanced magnetic resonance imaging revealed a pancreatic head lesion, suggestive of pancreatic cancer, with invasion and stenosis of the middle part of the common bile duct (Fig. 1). Multidisciplinary treatment was used to determine whether the patient had multiple metastases, biopsy was planned to clarify the pathology, and ERCP was used for jaundice reduction.

**Figure 2.**
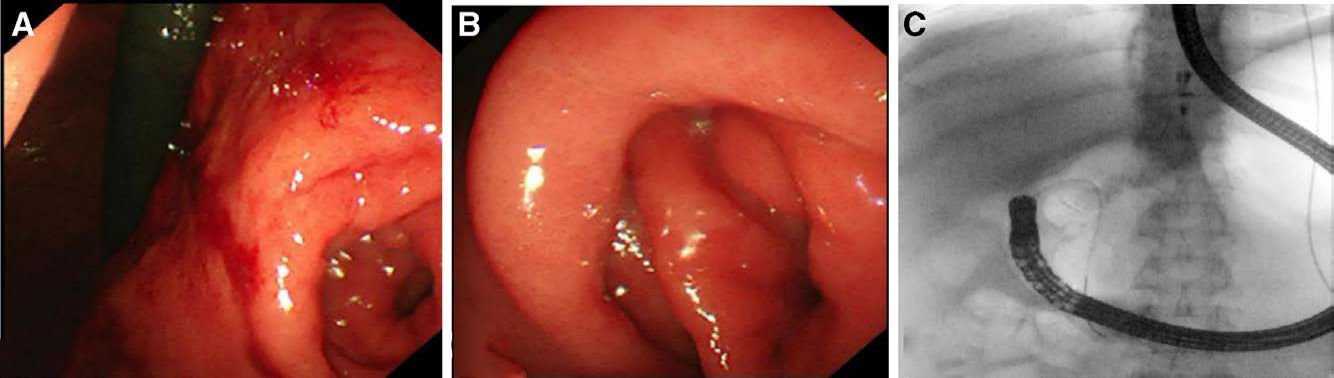
(A, B) The stomach is twisted, making it difficult to advance the endoscope. (C) Under the guidance of a cutting knife, the endoscope was inserted into the descending part of the duodenum.

**Figure 3.**
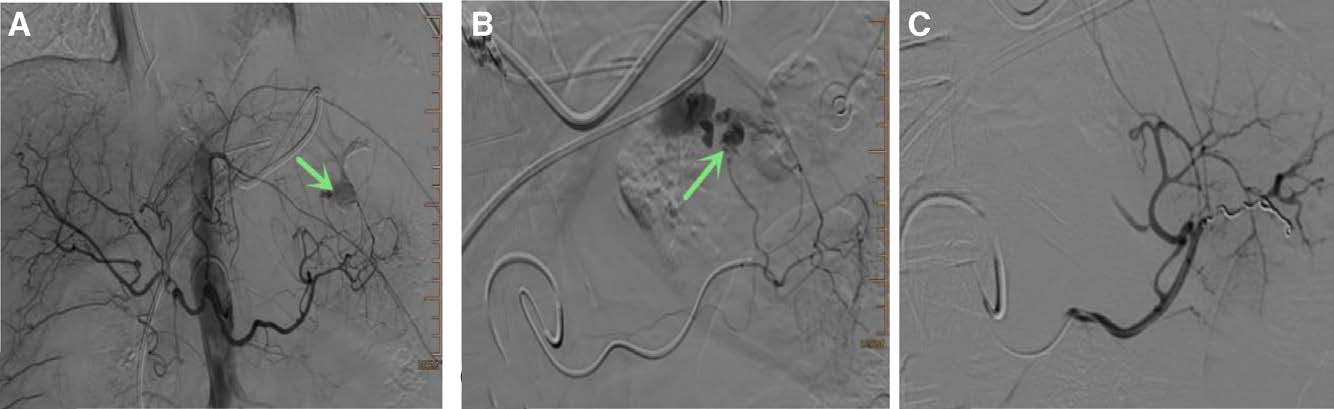
(A, B) Contrast agent extravasation was visible in the distal splenic artery branches (short gastric arteries). (C) After embolization of the distal splenic artery branches with coils, there was no contrast agent extravasation.

Biliary stent placement for jaundice reduction was planned using ERCP on December 24, 2024. The operator is highly experienced in ERCP and has completed nearly 20,000 procedures. During the procedure, the stomach cavity was significantly dilated, pylorus was twisted, and repeated attempts to pass through the pyloric orifice were unsuccessful. Finally, under the guidance of a cutting knife, the duodenoscope was inserted into the descending part of the duodenum, and the biliary stent was successfully placed (Fig. 2). The surgery lasted for 1 hour and 28 minutes. During the procedure, the patient’s heart rate increased to approximately 120 beats/min, and the lowest blood pressure was approximately 59/30 mm Hg. The hemoglobin level decreased from 140 to 102 g/L, and the hematocrit decreased from 42.7% to 30.8%. Rapid fluid resuscitation, vasopressors, and endotracheal intubation were administered. Bedside ultrasound revealed a large amount of fluid accumulation around the spleen and liver and ascites in the abdominal cavity, with an intact splenic capsule. Non-coagulating blood was aspirated from the abdominal cavity, suggesting abdominal bleeding. Further digital subtraction angiography (DSA) hemostasis was performed, revealing extravasation of the contrast agent from the distal splenic artery branches, indicating bleeding from the short gastric artery. The distal splenic artery branches were subsequently embolized with coils, with no extravasation of contrast medium (Fig. 3). After the procedure, the patient was transferred to the intensive care unit for further treatment and received blood transfusions, extubation, fluid replacement, and blood pressure elevation, after which the patient’s condition stabilized. The ventilator was removed 2 days later, and the patient was transferred back to the general ward for further treatment.

Two weeks later, the patient was discharged without any other complications.

## 3. Discussion

Splenic injury is a rare and unexpected complication after ERCP. The first case of splenic injury after ERCP was reported by Trondsen in 1989.^[4]^ Currently, there are only 34 reported cases of splenic injury following ERCP, with most involving splenic capsule tearing or subcapsular hematoma, and there have been no reports of isolated rupture of the splenic artery or short gastric artery with bleeding.^[1]^ The patient in this study experienced rupture and bleeding of the distal splenic artery (short gastric artery). In this case, the patient experienced tachycardia and low blood pressure during the procedure, which was initially misattributed to a tear in the cardia and did not receive sufficient attention. Owing to the patient’s hemorrhagic shock, there was no time to perform abdominal contrast-enhanced CT. Bedside ultrasound indicated the presence of blood around the spleen and liver, and nonclotted blood was aspirated during abdominal puncture, confirming intraabdominal bleeding. The patient was then directly subjected to DSA, which not only clarified the diagnosis but also served as an effective treatment option.

The mechanism underlying splenic artery injury and bleeding after ERCP remains unclear. The following 3 possible causes can be summarized through a review of the literature: First, the patient’s tumor invades the gastrointestinal tract and mesentery, causing excessive twisting and suspension of the stomach, making intubation difficult or violent, leading to tearing of the gastrosplenic ligament and rupture of the splenic artery.^[5]^ Second, a history of chronic pancreatitis or previous abdominal surgeries, such as liver transplantation, and left hepatectomy, plays an important role in splenic injury, with adhesion and fixation of the abdominal organs, making intubation difficult or excessive traction during position changes, which could lead to splenic artery injury.^[6,7]^ Additionally, the patient was in a prone position, and splenic injury occurred during postoperative handling. Considering the characteristics of this patient, the first cause is more likely.

This complication lacks specific clinical manifestations, often presenting with sudden abdominal pain, which overlaps with post-ERCP abdominal pain symptoms and is prone to misdiagnosis. Bedside abdominal ultrasound examination is very important, and enhanced abdominal CT examination should be carried out. Regarding the treatment of splenic injury after ERCP, the splenectomy rate caused by ERCP is as high as 80%, and only a small number of cases can be successfully treated.^[8]^ However, in this case, direct DSA angiography was performed, which is both a diagnostic method and an effective treatment for avoiding splenectomy. Splenic injury or splenic artery bleeding is an unexpected and extremely rare complication of ERCP and distal splenic artery branch embolization with DSA is a possible treatment approach.

## Author contributions

**Visualization:** Zhiqiang Huang.

**Writing – original draft:** Zhiqiang Huang.

**Writing – review & editing:** Yunsheng Qin.

## References

[1] Kourdakis DS Deftereos SP. Endoscopic retrograde cholangiopancreatography induced splenic injury: comprehensive analysis and new perspectives based on a case report. Ther Adv Gastrointest Endosc. 2024;17:26317745231223312. 38223215 10.1177/26317745231223312 PMC10787536

[2] Manoharan D Srivastava DN Gupta AK Madhusudhan KS. Complications of endoscopic retrograde cholangiopancreatography: an imaging review. Abdom Radiol (NY). 2019;44:2205–16. 30809695 10.1007/s00261-019-01953-0

[3] Momani LA Karar S Shipley LC Locke A Swenson J. Endoscopic retrograde cholangiopancreatography-induced splenic injury in a patient with sleeve gastrectomy. J Investig Med High Impact Case Rep. 2018;6:2324709618779417. 10.1177/2324709618779417 PMC6024491 29977934

[4] Trondsen E Rosseland AR Moer A Solheim K. Rupture of the spleen following endoscopic retrograde cholangiopancreatography (ERCP). Case report. Acta Chir Scand. 1989;155:75–6. 2929211

[5] Paredes AH Williams AM Vertrees AE Womeldorph C. Splenic laceration following ERCP. Endoscopy. 2013;45(Suppl 2 UCTN):E221–2. 23945920 10.1055/s-0033-1344021

[6] Gaffney RR Jain V Moyer MT. Splenic injury and ERCP: a possible risk for patients with advanced chronic pancreatitis. Case Rep Gastroenterol. 2012;6:162–5. 22679404 10.1159/000337499 PMC3364081

[7] Lubikowski J Piotuch B Przedniczek M Sabadosh R Wojcicki M. Looking for a cause of the spleen rupture following endoscopic retrograde cholangiopancreatography. Eur J Gastroenterol Hepatol. 2020;32:129–30. 31790006 10.1097/MEG.0000000000001554

[8] Yue P Meng WB Li X. Rare complications related to endoscopic retrograde cholangiopancreatography and its management. Chin J Pract Surg. 2018;38:938–41.

